# Effect of Adiantum Capillus veneris Linn on an Animal Model of Testosterone-Induced Hair Loss 

**Published:** 2014

**Authors:** Maryam Noubarani, Hossein Rostamkhani, Mohammad Erfan, Mohammad Kamalinejad, Mohammad Reza Eskandari, Mohammad Babaeian, Jamshid Salamzadeh

**Affiliations:** a *Department of Pharmacology and Toxicology, School of Pharmacy, Zanjan University of Medical Sciences, Zanjan, Iran .*; b *School of Pharmacy, Shahid Beheshti University of Medical Science, Tehran, Iran. *

**Keywords:** Adiantum capillus-veneris linn, Anderogenetic alopecia, Testosterone, Hair growth

## Abstract

Androgenetic alopecia is the most common form of hair loss in men. The present study was designed to evaluate the hair growth-promoting activity of a preparation of the Adiantum capillus-veneris Linn. (A. capillus-veneris) on albino mice using a testosterone-induced alopecia model. Five groups of albino mice were studied: (A) Testosterone solution only (n=6); (B) Testosterone + Finasteride solution (2%) (n=6); (C) Testosterone + vehicle (n=6); (D) Testosterone + A. capillus-veneris solution (1%) (n=6); (E) intact control (n=2, without testosterone). Alopecia was induced in all intervention groups by testosterone 1.0 mg subcutaneous. A. capillus-veneris solution was applied topically to the back skin of animals in the respective group. Hair growth was evaluated by visual observation and histological study of several skin sections via various parameters as follicle density (number of follicles/mm) and anagen/telogen ratio. After 21 days, a patch of diffuse hair loss was seen in animals received testosterone while animals treated with A. capillus-veneris showed less hair loss as compared to those treated with testosterone only. The follicular density observed in the A. capillus-veneris-treated group was 1.92 ± 0.47, compared to 1.05 ± 0.21 in testosterone-group and 2.05 ± 0.49 in finasteride-treated animals. Anagen/telogen ratio was significantly affected by A. capillus-veneris, which was 0.92 ± 0.06 as compared with 0.23 ± 0.03 and 1.12 ± 0.06 for testosterone and finasteride treated groups, respectively. According to visual observation and quantitative data (follicular density and anagen/telogen ratio), A. capillus-veneris was found to possess good activity against testosterone-induced alopecia.

## Introduction

Androgenetic alopecia or simply “balding”, the most common form of hair loss in men, involves the progressive hair loss, in response to circulating androgens. It may also occur in women (1). Although, there is racial variation in the incidence of androgenetic alopecia, it affects at least 50% of men by the age of 50 years, and up to 70% of all males in later life (2). Prevalence in women has wide variation ranging from six percent in women aged under 50 years to 30-40% of women aged >70 years (3). Treatment of androgenetic alopecia has been one of the major concerns for cosmetologists and dermatologists. Over the centuries, a wide range of remedies have been suggested for androgenetic alopecia and currently treatments include wigs and hairpieces, surgery, hormone action modifiers, and non-hormonal therapy. Pharmacological therapies are based on understanding of the mechanism of action of androgen in hair follicle (4). Although, only Minoxidil and Finasteride currently have US Food and Drug Administration (FDA)–approval for treatment of androgenetic alopecia, however, because of adverse reactions, their usage is limited (5). Use of natural products has been quiet common in hair care industry and the search for natural products is being continuously promoted (6). Hair growth-promoting effects of *Abrus precatorius *(7), *Cuscuta reflexa *(8), *Citrullus colocynthis *(9) and *Eclipta alba *(10) was previously reported in different studies. *Adiantum *is one of the herbs with the traditional claims of hair growth promotion in topical use (11). *Adiantum *is a large genus of ferns which are widely distributed throughout the world. Amongst the nine species of this plant, *Adiantum capillus-veneris Linn*. (*A. capillus-veneris Linn*) is the only species found in Iran (12, 13). *Adiantum capillus-veneris *has been shown to exhibit antifungal (14), antibacterial (15), antiviral (16) anti-inflammatory, anti-nociceptive (17) and antioxidant activities (18). It is an important drug in ancient literature of Unani system of medicine which has widely been used in patients with urolithiasis and its decoction was claimed to have litholytic activity by several Unani physicians (19). According to the claims in Iranian traditional medicine confirming hair growth promotion by *A. capillus-veneris*, the present investigation was carried out to evaluate effect, using a pre-clinical experimental testosterone-induced alopecia applying an animal model. 

## Experimental

*Plant material and Extraction*

Dried arial parts of the plant *A. capillus-veneris *were purchased from the local market in Tehran, Iran. This plant is collected from the forest of Northern Iran provinces, Gilan and Mazandaran, during May to June. A botanist approved the taxonomy of plant and confirmed its species. The dried plant was powdered and extracted with maceration in ethanol (95% v/v). The extract was freed from the solvent under vacuum in a rotary evaporator. The yield of the ethanolic extract was 3% w/w.

*Animals*

The protocol for experimentation was approved by the Institutional Animal Ethics Committee of Shahid Beheshti University of Medical Sciences (Tehran, Iran). Male albino mice (2–3 months) were kept in cages at the room temperature and fed with food and water. 

*Sample preparation*

The ethanol extracts of *A. capillus-veneris *were incorporated into solution in concentration of 1% (w/v). Solution was made with ethanol base and adding propylene glycol in proportion of 90:10. The extract was then incorporated into prepared solution.

*Testosterone test solution*

Testosterone solution was prepared as a suspension in the vehicle (ethanol/propylenglycol 90:10).

*Finasteride solution*

The 2% standard finasteride solution was prepared in vehicle (ethanol/propylene glycol, 90:10).

*Treatments and animals for study*

The method reported by Matias *et al. *(20) was followed with slight modification. A similar procedure was used by Pandit *et al. *(8) and Dhanotia *et al. *(9). In brief, twenty six adult male albino mice 2–3 months of age were randomly allocated in five groups of six animals per group (except group E with two mice). The various groups were treated as follows: (A) Testosterone solution only; (B) Testosterone + Finasteride solution (2%); (C) Testosterone + vehicle; (D) Testosterone + *A. capillus-veneris *solution (1%); (E) intact control (without testosterone). Mice in all groups except group E were administered testosterone (1 mg) subcutaneously (SC). Animals of groups B, C and D were also received topical application of finasteride (2%), 0.5 mL vehicle, and *A. capillus-veneris *solution (1%), respectively, on back skin once a day for 21 days. 

*Qualitative evaluation of hair growth*

The difference in growth of hair in each group was evaluated by visual observations and was recorded by photographs after 21 days.

*Quantitative evaluation of hair growth*

The skin of each animal from the dorsal area was dissected after shaving the long hair and preserved in 10% formalin. After fixation, vertical sections of the skin were prepared. The sections were stained with hematoxylin and eosin. The sections were then observed for different parameters for evaluating hair growth. The number of hair follicles in 2 mm area was recorded and reported as follicular density (number of follicles/mm). The number of follicles in anagen phase (active growth phase) and those in telogen phase (resting phase) were also counted, and anagen/telogen ratios were determined.

*Statistical analysis*

Data are reported as mean ± SD. Data was analyzed by one-way analysis of variance (ANOVA) followed by the Tukey’s HSD as the post hoc test. P < 0.05 was considered as significance level. 

## Results

*Qualitative study*

**Figure 1:**
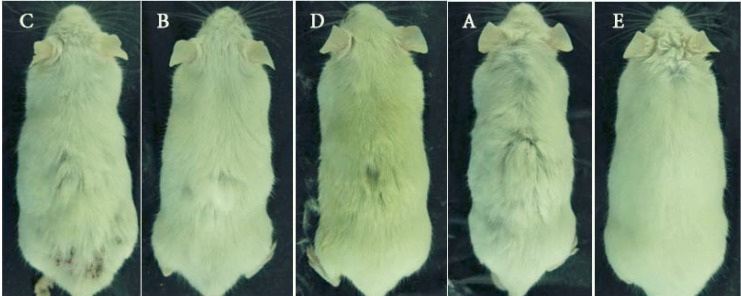
Comparison of hair growth/loss patterns in various groups after 21 days: (C) animal treated with testosterone plus vehicle, (B) animal treated with testosterone plus 2% finasteride, (D) animal treated with testosterone plus 1% *A. capillus veneris Linn*, (A) animal received testosterone, (E) control animal

The animals of groups A and C showed a patch of diffuse hair loss. Loss of hair from dorsal portion of rat was clearly visible after a 21-day treatment with testosterone. After 21 days, the visual observations suggested that the animals treated with *A. capillus-veneris *showed less hair loss as compared to those received testosterone. The hair growth pattern in *A. capillus-veneris-*treated groups was comparable to that for the standard drug finasteride (Figure 1).

*Quantitative study*

**Figure 2:**
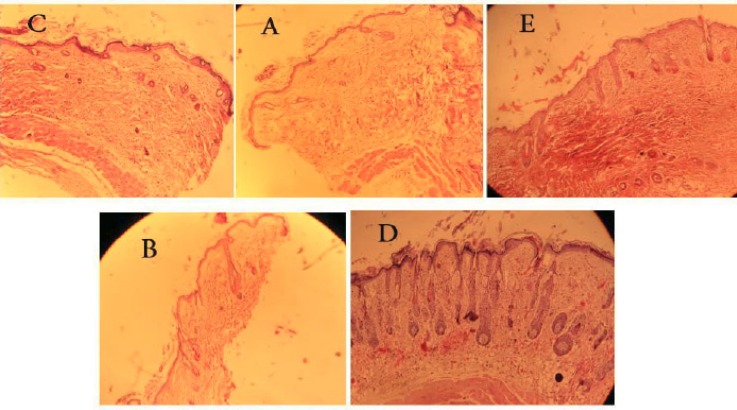
Histology of skin sections: (C) Skin of animal treated with Testosterone and vehicle, (A) Skin of animal treated with testosterone, (E) Skin of intact animal, (B) Skin of animal treated with testosterone and finasteride solution, (D) Skin of animal treated with testosterone and *A. capillus veneris Linn*

Microscopic photograph of skin sections of animals treated with testosterone and those with testosterone plus vehicle revealed that testosterone treatment caused miniaturization of hair follicles. The follicles showed bulbous appearance and were short. The effect of testosterone on miniaturization of hair follicle was blocked by administration of topical finasteride and *A. capillus-veneris *in B and D animal groups, respectively (Figure 2). The histological observations showed an increase in the length as well as number of hair follicles in *A. capillus-veneris-*treated group. The follicular density (number of follicles/mm) was calculated. The follicular density observed in the *A. capillus-veneris-*treated group was 1.92 ± 0.47, whereas it was 1.05 ± 0.21 in testosterone-treated animals and 2.05 ± 0.49 in finasteride-treated animal (Table 1).

**Table 1:** Follicular density and anagen/telogen ratio in sections of skin of different groups of study animals

**Anagen/telogen ratio**	**Follicular density (no./mm),mean ± SD **	**Number**	**Group**
1.35 ±0.05	2.36 ± 0.34	2	Normal
0.23 ± 0.03*	1.05 ± 0.21*	6	Testosterone (s.c.)
0.25 ± 0.05*	1.18 ± 0.41*	6	Testosterone (s.c.) + vehicle (topical)
1.12 ± 0.06**	2.05 ± 0.49**	6	Testosterone (s.c.) + 2% Finasteride solution (topical)
0.92 ± 0.08**	1.92 ± 0.47**	6	Testosterone (s.c.) + 2% *A. capillus veneris Linn*. sotion ( topical)

Values represent mean±SD

*p<0.005, significance vs control

**p<0.05, , significance vs testosterone (s.c.)

**Figure 3:**
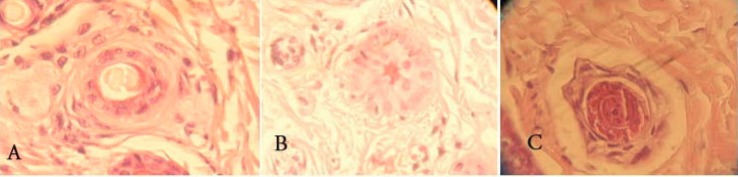
Histological aspect of follicles: A) terminal anagen with inner and outer root sheath, B) terminal catagen with thickening of the basal membrane and C) terminal telogen with wrinkling of the inner root sheath

The cyclic phase of hair follicles (anagen, telogen) was determined, and the anagen/telogen ratio was calculated. Anagen follicles were recognized when fully developed inner and outer root sheaths were identified, with no signs of apoptosis in the outer root sheath (Figure 3A). Follicles with central wrinkling of the hair canal (tricholemal keratinization) were considered as telogen (Figure 2B). Follicles with thickening of the basal membrane or apoptotic cells were determined as catagen (Figure 3C). Because catagen hairs inevitably become telogen hairs, catagen was included in the telogen phase when calculating anagen/telogen ratio.

The number of follicles in anagen phase was considerably increased and fewer follicles in telogen phase were observed in *A. capillus-veneris-*treated animals. Anagen/telogen ratio was significantly affected by *A. capillus-veneris, *which was 1.12 ± 0.06 as against 0.23 ± 0.03 for testosterone-treated animal and 0.92 ± 0.06 for finasteride-treated animals (Table 1). The predominance of hair follicle in anagenic growth phase indicates the reversal of androgen induced hair loss in *A. capillus-veneris*-treated animals. As it is evident from the above data, the activity of *A. capillus-veneris *is comparable with finasteride.

## Discussion

Androgenetic alopecia is a heritable and androgen-dependent disorder occurs in a defined pattern. Testosterone is required, along with a genetic predisposition, for androgenetic alopecia (21). Hair follicles are the targets for androgen-stimulated hair follicle miniaturization, leading to replacement of large, pigmented hairs (terminal hairs) by barely visible, depigmented hairs (vellus hairs). The result is a progressive decline in visible scalp hair density. The current model for androgen action in the hair follicle focuses on the mesenchyme derived, regulatory dermal papilla at the base of the follicle. This responds to the circulating hormones and coordinates the rest of the follicular cells by altering the paracrine signals it produces (22). 

According to our knowledge the current study is the first report on hair growth potential of *A. capillus-veneris *we have used the mouse as a model for evaluation of hair growth activity of this plant by the prepared biopsies. While new methods like trichologic follow-up, video-dermoscopy, pull test and TrichoScan are available in evaluating hair growth activity, biopsy as the oldest method is still helpful tool. Biopsy is an easy procedure, associated with little pain, providing the most anatomical and histopathological features of hair follicles (23).

Testosterone induced alopecia, was counteracted when finasteride was administered simultaneously to the mice. In hair follicles, the androgen exerts its effect either directly or after conversion by an enzyme, 5α reductase, to dihydrotestosterone, which is a more potent androgen that binds to androgen receptors in hair follicles (24). Finasteride is an inhibitor of 5α-reductase and has been used in other testosterone-induced alopecia experiments (7-9). The alopecia was not evident in animals that were treated with *A. capillus-veneris *along with testosterone. Besides visual observation, quantitative data (follicular density and anagen/telogen ratio) also suggest inhibition of androgenic activity by *A. capillus-veneris*. Thus, *A. capillus-veneris *is considered a useful preparation for topical use in commercial formulations for androgenic alopecia and other androgen related disorders. 

Phytochemical screening of *A. capillus-veneris *has revealed existence of an array of compounds including triterpenes, flavonoids, phenylpropanoids and carotenoids in this plant (25). Presence of flavonoids can provide an antioxidant activity for *A. capillus-veneris *(18). On the other hand, Kim *et al. *has reported that testosterone may induce hair loss by apoptosis of hair follicles rather than through the androgen metabolic pathway (26). Therefore, it could be suggested that flavonoids could participate in hair growth potential of this plant. 

This preclinical study introduces an initial concept for hair growing potential of *A. capillus-veneris*. Further studies investigating antianderogenic mechanism of this plant as well as human studies will be complementary to this study.
